# Lessons learned: drive-through COVID-19 clinic testing during an adaptive epidemic response and a point-of-care test assessment of a computer-read rapid lateral flow immunoassay with fluorescence-based detection

**DOI:** 10.1099/jmm.0.001875

**Published:** 2024-09-02

**Authors:** Leah Rankine-Wilson, Teresa Oncken, Irshan Basrewan, Courtney Jeffery, Todd M. Pryce, Rebecca Wake, Aus A. L. Molan, T. F. Paton, Tim J. J. Inglis

**Affiliations:** 1 Department of Clinical Microbiology, PathWest Laboratory Medicine WA, Fiona Stanley Hospital, Murdoch, Australia; 2 Department of Microbiology and Immunology, The University of British Columbia, Vancouver, Canada; 3 Department of Microbiology, PathWest Laboratory Medicine WA, Nedlands, Australia; 4 PathWest Corporate, PathWest Laboratory Medicine WA, Nedlands, Australia; 5 School of Medical and Health Sciences, Edith Cowan University, Joondalup, Western Australia, Australia; 6 Harry Perkins Institute for Medical Research, Nedlands, Western Australia, Australia; 7 School of Medicine, University of Western Australia, Crawley, Western Australia, Australia; 8 Western Australian Country Health Service, Curtin University Campus, Bentley, Western Australia, Australia

**Keywords:** border restriction, conformity study, COVID-19, drive-through clinic, emergency response, PCR, rapid antigen test, SARS-CoV-2, trial, Western Australia

## Abstract

**Background.** The COVID-19 pandemic demonstrated a need for robust SARS-CoV-2 test evaluation infrastructure to underpin biosecurity and protect the population during a pandemic health emergency.

**Gap statement.** The first generation of rapid antigen tests was less accurate than molecular methods due to their inherent sensitivity and specificity shortfalls, compounded by the consequences of self-testing. This created a need for more accurate point-of-care SARS-CoV-2 detection methods.

**Aim.** Here we present the lessons-learned during the COVID-19 emergency response in Western Australia including the detailed set-up, evaluation and operation of rapid antigen test in a state-run drive-through sample collection service during the COVID-19 pandemic after the strict border shutdown ended.

**Methods.** We report a conformity assessment of a novel, second-generation rapid antigen test (Virulizer) comprising a technician-operated rapid lateral flow immunoassay with fluorescence-based detection.

**Results.** The Virulizer rapid antigen test demonstrated up to 100% sensitivity (95% CI: 61.0–100%), 91.94% specificity (95% CI: 82.5–96.5%) and 92.65% accuracy when compared to a commercial PCR assay method. Wide confidence intervals in our series reflect the limits of small sample size. Nevertheless, the Virulizer assay performance was well-suited to point-of-care screening for SARS-CoV-2 in a drive-through clinic setting.

**Conclusion.** The adaptive evaluation process necessary under changing pandemic conditions enabled assessment of a simple sample collection and point-of-care testing process, and showed how this system could be rapidly deployed for SARS-CoV-2 testing, including to regional and remote settings.

## Data Summary

The authors confirm all supporting data, code and protocols have been provided within the article or through supplementary data files.

## Introduction

The COVID-19 (coronavirus infectious disease 2019) pandemic, caused by the virus SARS-CoV-2 (severe acute respiratory syndrome coronavirus 2), made its mark on global public health with over 776 million confirmed cases, and around seven million deaths [1]. In March 2020, the state government of Western Australia (WA) closed its borders to non-essential travel and introduced mandatory quarantine for all interstate and international arrivals. The spread of COVID-19 was managed at both federal and state levels, including testing, contact tracing and quarantine (TTIQ) programmes. PathWest Laboratory Medicine WA, Western Australia’s state-run pathology service, was responsible for quarantine facility testing from the early stages of the shutdown period.

**Fig. 1.**
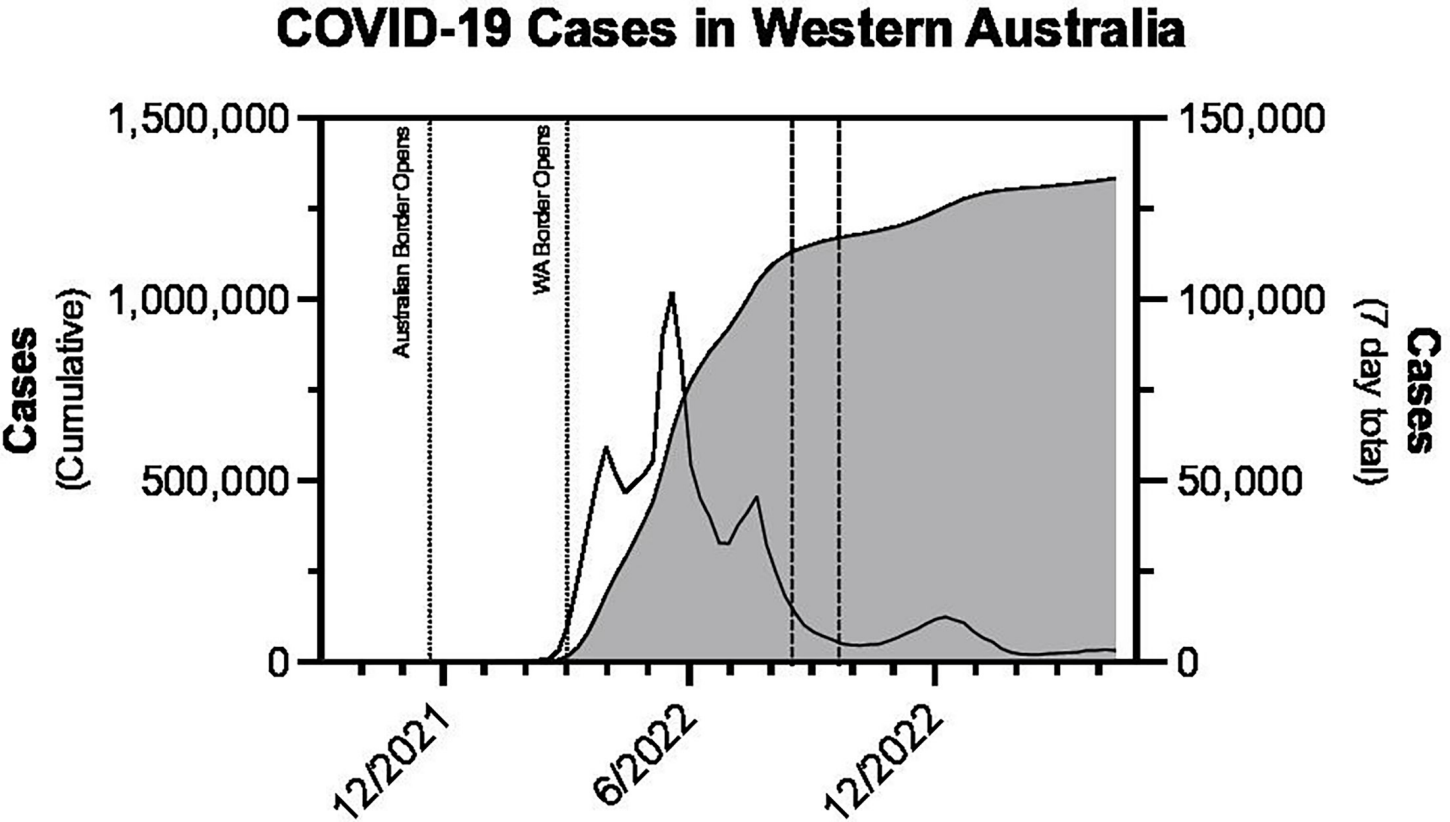
Cumulative (left) and weekly total (right) registered COVID-19 cases in Western Australia and notable dates 2020-2023. Dashed lines mark the beginning and end of the Virulizer conformity trial. Data retrieved from [3].

The state of Western Australia has a total land area of 2.53 million square kilometres: approximately one third of Australia’s land mass. With a population density of 0.89 people per square kilometre and 79% of the population living in the metropolitan region of Perth, WA is considered one of the least densely populated states in the world [2]. Consequently, WA’s COVID-19 emergency response exploited its geographical isolation through quick introduction of border restrictions which led to initial containment and elimination of the virus from the population (Fig. 1) [3]. While global case numbers increased significantly, curtailment of new infections in WA enabled the supply of emergency testing capacity to closely match the level of public health demand until planned relaxation of border controls.

Previous studies described drive-through testing centres (DTC) as a suitable solution for high-volume specimen collection and testing subject to adequate physical distancing/protective equipment use between the collector and patient [4,5]. When WA borders reopened in March of 2022, a COVID-19 negative (not detected) self-administered rapid antigen test (RAT) or polymerase chain reaction (PCR) result was required prior to travel, return to work after illness, or routinely for FIFO and hospital workers [6]. The state’s COVID-19 testing arsenal consisted of public hospital-based walk-in clinics, privately operated DTC’s and several publicly operated DTCs in the Perth metropolitan area. The DTC located at Murdoch University (MDTC) was set up to increase the COVID-19 PCR collection capacity in order to relieve pressure on hospital-based COVID-19 clinics. The MDTC was the largest drive-through collection centre in the state and had a maximum output of 1,300 sample collections per day. PCR testing was supplemented with self-reported home testing RATs, which were regularly analysed for effectiveness. These have proved to be an inconsistent screening tool due to the lack of standardisation, since patients are able to self-administer the test and determine results without supervision of prior training or specimen collection [7,9]. Accurate self-diagnosis by RAT is further limited by the colorimetric indicators, which can produce faint positive lines that are undetectable by the naked eye, and ultimately increasing the risk of further infection spread. In addition, when self-reporting RAT results for return-to-work, social or school purposes, personal concerns about economic, social, or academic welfare may further contribute to SARS-CoV-2 spread [10,12].

The Virulizer SARS-CoV-2 detection system addressed the absence of a locally based biotechnology industry capable of RAT manufacture. A locally based manufacturer adapted workplace health and safety equipment (Alcolizer) into a small, portable device that could be operated by a trained technician at the point of collection to detect SARS-CoV-2 in a minimally invasive oral fluid specimen. This combination of ease-of-use, lateral flow immunoassay and fluorescence-based detection method was considered an improvement on nasal-based rapid antigen testing.

This paper presents a proof-of-concept study that examines two interconnected aspects of Western Australia’s COVID-19 pandemic response: the conformity assessment of a novel rapid antigen test and the implementation of emergency response measures, including the establishment of drive-through testing clinics. Our study provides insights into the challenges and opportunities encountered during the pandemic, focusing on the evaluation of new point-of-care testing technology during a rapidly evolving public health emergency. While the small sample size limits definitive conclusions about specific test performance, this work offers valuable lessons for future pandemic preparedness and highlights the potential of adaptive testing approaches during a fluid emergency epidemic response.

## Methods

### Ethics and research governance

The study, known as Project CRATE (COVID Rapid Antigen Test Evaluation) and its subcomponents, was approved by the Sir Charles Gairdner and Osborne Park Hospital Group Research Ethics Committee (PRN: RGS0000005374) and the PathWest Laboratory Medicine Research Governance process, as a subproject of Project ADEPT (Adaptive Diagnostics for Emerging Pathogenic Threats, 2021/GNT2012074), which was funded by the Australian National Health and Medical Research Council (NHMRC) under the Ideas research grant programme. Patient specimens were collected with the participant’s written, informed consent on the understanding that Virulizer assay results would be used only for test performance analysis and not used to determine any clinical or public health action.

### Drive-through centre set-up and management

#### MDTC staff training and site setup

All MDTC staff underwent a half day of training before starting on-site work. Full-set clinical personal protective equipment (PPE) was mandatory for MDTC staff and was worn no longer than 5 h at a time in accordance with World Health Organization (WHO) recommendations [13]. Demonstration of optimal saliva collection for the Virulizer assay protocol was conducted by company (Alcolizer, Balcatta, WA) staff during the initial stages of the proof-of-concept and calibration series. Virulizer staff were consulted only to demonstrate the optimal user protocol for sample collection during set-up, and thereafter to provide technical support for the detection platform in-field installation. They were excluded from participant recruitment, data collection, analysis, or interpretation of results in accordance with the approved study protocol, ensuring the independence and objectivity of the study. All other onsite training, including the use of the Virulizer equipment platform and specimen collection, and workflow troubleshooting, was provided by the named authors. Standardized PathWest DTC protocols were used for cleaning, material handling and specimen packaging.

**Fig. 2.**
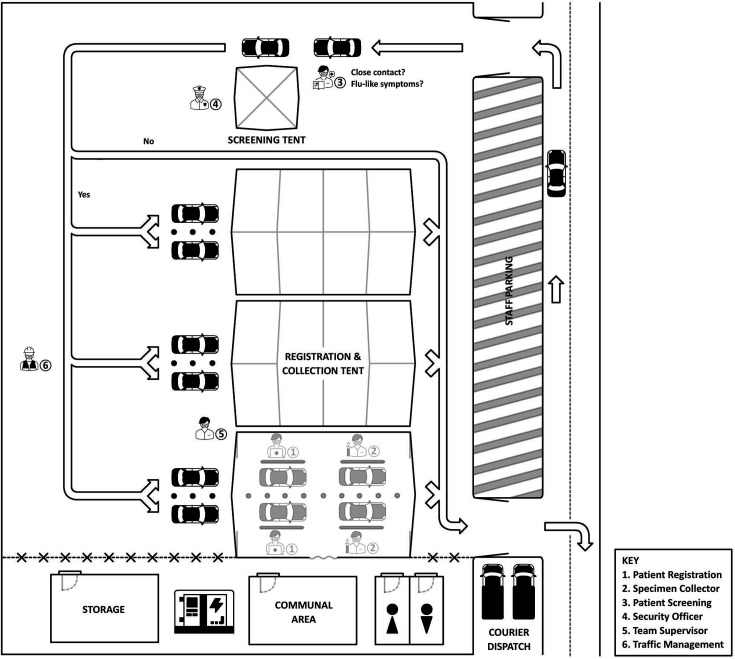
Initial drive-through centre schematic for COVID-19 testing with separated patient registration and testing posts. Arrows indicate the flow of traffic through the clinic. Patients entered from the adjoining street and were screened for eligibility [3] before being redirected to appropriate lane [6]. Each lane was manned by one registration [1] and one sample collection [2] team member with separate stations. Security personelle [4] were located at point of entrance. Site maintenance facilities were located in a restricted, PC1 area.

The site chosen for the MDTC was accessible to the public, had space for safe traffic management without overflow onto high vehicle traffic flow areas nearby, and a screening area to request, inform and consent participants. MDTC arrivals were accepted for PCR testing if they had any symptom associated with COVID-19. Close contacts not displaying symptoms were provided free first-generation RATs at the screening tent before exiting the testing area. The MDTC was close to a large PathWest microbiology laboratory. The MDTC site was re-surfaced prior to use with recycled asphalt to reduce dust aerosols and provide a level surface. Three large marquees, each 15 metres long were erected with two lanes per marquee and two stations per lane (Fig. 2). Operating hours were 08 : 00 – 20 : 00, 7 days a week. A screening tent was installed near the site entrance for initial patient assessment.

### SARS-CoV-2 RNA testing

A combined nasopharyngeal and oropharyngeal swab (naso-oropharyngeal) was collected from each patient for SARS-CoV-2 PCR assay immediately before collection of the RAT sample . The swab was inoculated into 3 mL of transport media (UTM-RT, Copan, Brescia, Italy). All samples for PCR were tested using a SARS-CoV-2 PCR assay according to the manufacturer’s instructions (COBAS, Roche Diagnostics, Basel, Switzerland). The PCR assay targets *ORF1a/b* (a non-structural region unique to SARS-CoV-2) and *E-gene* (a structural protein envelope gene for pan-sarbecovirus detection). A 600 mL aliquot of sample was transferred to a secondary tube (COBAS Omni) then capped and thermally treated at 75 °C for 15 min as described previously [14]. Samples were transferred to the COBAS 6800 instrument (Roche). Positive test results for *ORF1a/b* and/or E-gene were taken as evidence of SARS-CoV-2 detection.

### Quantitative standards, external controls, and analysis

Quantitative standards were prepared from the first WHO International Standard for SARS-CoV-2 (SARS-CoV-2 IS, NISBSC code 20/146), supplied as 7.70 log_10_ IU mL^−1^ (National Institute for Biological Standards and Control, Hertfordshire, UK) as previously described [15]. Briefly, the standard was reconstituted with 0.5 mL of phosphate buffered saline per manufacturer’s instructions. The standard was then serially diluted ten-fold in a naso-oropharyngeal matrix. This matrix consisted of pooled naso-oropharyngeal samples from samples previously tested as negative for SARS-CoV-2 using the COBAS PCR assay. Seven standards were prepared over the range of 0.70 to 6.70 log_10_ IU mL^−1^. Each standard was tested in triplicate by COBAS. The mean cycle of quantification (*C_q_*) value at each concentration was used to calculate *ORF1a/b* and *E-gene* standard curves and regression. At least two positive replicates at each dilution were included in the standard curve. Regression formulas were used to calculate the *ORF1a/b* and *E-gene* log_10_ IU mL^−1^ for all positive samples.

### Participant recruitment

MDTC attendees were invited to volunteer for the CRATE study after screening staff ensured each participant met the trial inclusion criteria (aged between 16 and 80 years, no food or drink consumption other than water in the 15 min before testing). All test cartridges were placed in the Virulizer equipment unit in under 6 min from completion of sample collection.

#### Trial inclusions and exclusions

Eleven of the 82 collections failed to convert the oral fluid collector (OFC) indicator panel to blue within the defined 2 min of sampling. Each OFC uses a pH-based colour indicator that changes from white to yellow to blue according to the amount of absorbed saliva. A full blue colour change indicated ≥1 mL of saliva had been collected, while yellow indicated the collection had initiated but not yet reached sufficient volume. As all 11 failed specimens produced a colour change from white to yellow, the decision was made to analyse the results in two sets: one set containing all tested samples and another that excluded results from OFCs that failed to fully convert to blue.

#### SARS-CoV-2 detection using Virulizer platform

**Fig. 3.**
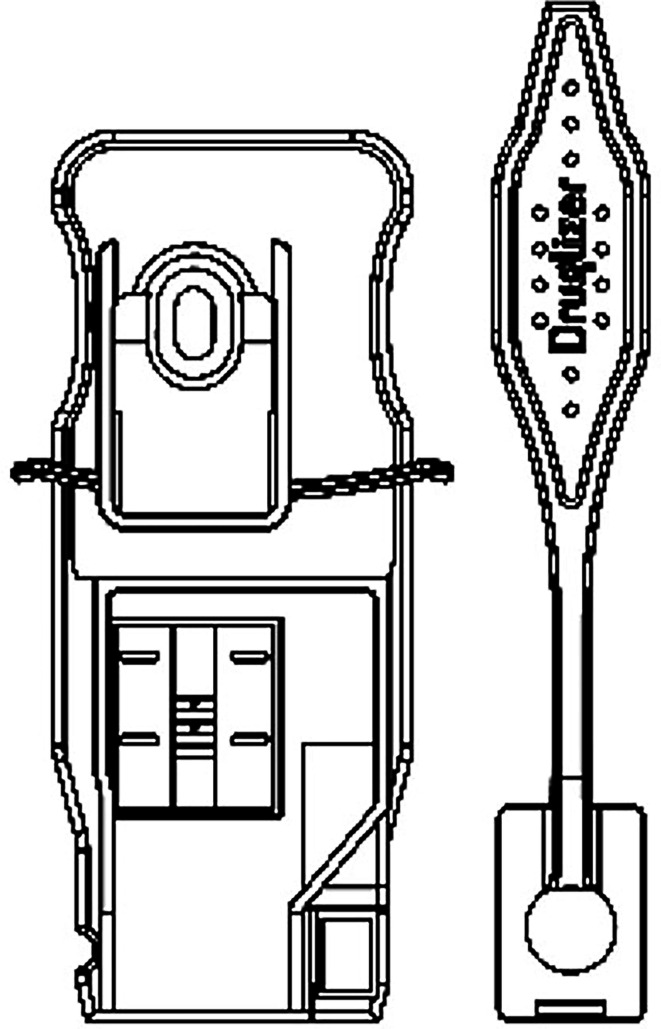
Virulizer cartridge (left) and oral fluid collector (OFC) (right).

All the components of the Virulizer test kit (cartridge, oral fluid collector, lysis buffer, dropper) were supplied and manufactured by Alcolizer Technology (Balcatta, WA, Australia). The Virulizer device uses lateral flow immunoassay and fluorescence-based detection of SARS-CoV-2 nucleocapsid protein from approximately 500 µL of saliva to produce a qualitative result within 6 min of sample collection. The SARS-CoV-2 test kit contains an OFC, a dropper tube containing lysis buffer and a SARS-CoV-2 test cartridge. Saturated collectors were placed into the dropper tube then four drops of liquid sample were dispensed onto the test cartridge after a short incubation. The cartridges comprise a lateral flow strip containing lanthanide-based upconversion nanoparticles (UCNPs) and a programmable test-specific electronic chip that are inserted into the Virulizer instrument for optical analysis of lateral flow immunoassay (Fig. 3). The Virulizer SARS-CoV-2 test cartridge follows general lateral flow immunoassay convention via capillary action of antigen along a nitrocellulose membrane to a capture test line. However, it differs to typical lateral flow strips in that it uses UCNPs as the antigen-capturing conjugate. Once captured along a test line, the UCNPs are excited by a low-energy infrared laser inside the Virulizer instrument to produce high-energy, background-free fluorescence emissions which is enhanced on an image sensor and processed for signal strength. An increase in antigen presence relates to an increase in signal [16].

### Data analysis

Statistical analyses were performed in Excel (Microsoft, Redmond, WA, USA) and MedCalc Statistical Software v 15.4 (MedCalc Software Ltd, Ostend, Belgium). The graphs were prepared using GraphPad Prism version 8.0.0 for Windows (GraphPad Software, San Diego, California USA). A supplementary file contains formulas used for data analysis (Table S1, available in the online Supplementary Material) and Interpretations of Cohen’s Kappa (Table S2).

## Results

### Drive-through site operation

The MDTC allowed collection of ~1,300 swabs per day in a single 12 h shift, though at peak capacity was stretched to an estimated 1,500 swabs collected. An online patient pre-registration system (PRS) that was accessible to the public, was implemented in April 2022 to allow collection of patient demographic information. Patients were directed to register prior to presenting at a collection centre to reduce laboratory registration time so that staff could access, cross-check patient information and obtain their consent before specimen collection. This allowed funnelling of registered patients into a designated lane to improve traffic flow and patient throughput. The integrity of patient information in the data chain was secured by direct transfer from the PRS into the laboratory information system (LIS), rather than by manual transcription from written request forms.

**Fig. 4.**
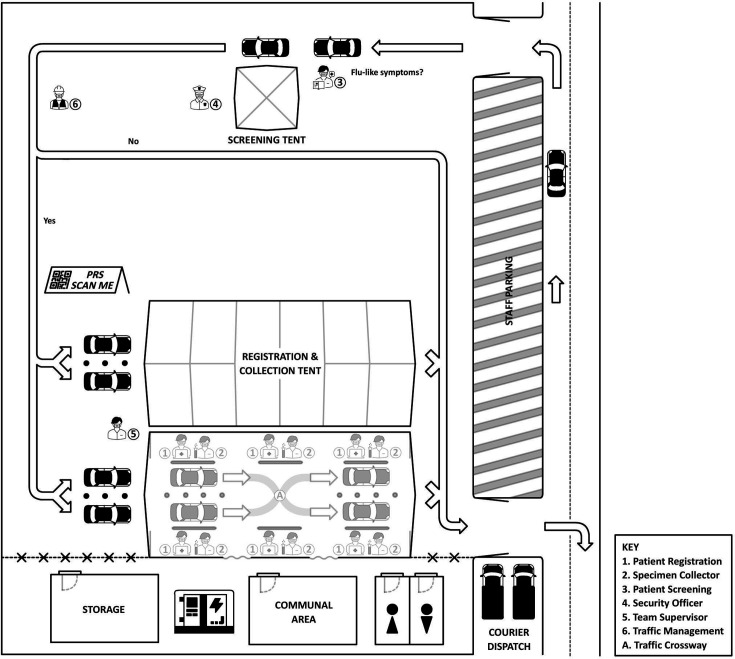
Adapted drive-through centre schematic for COVID-19 testing utilizing online patient registration system (PRS) in the pre-clinical wait area. Arrows indicate the flow of traffic through the clinic. Patients entered from the adjoining street and were screened for eligibility [3] before being redirected to appropriate lane [5]. Each station had registration [1] and collection [2] capabilities manned by teams of two with the ability to move freely as well as redirect traffic through the crossway (A).

Fluctuating test demand led to changes in the MDTC site layout during its operational life. A longer marquee protected staff and participants when weather conditions deteriorated with the change of season. The inclusion of a crossover space between sample collection lanes enabled traffic flow in the event of vehicle breakdown or general holdups (Fig. 4, Item A). Traffic flow and traffic-related incident management was improved by these changes, and the authors recommend dedicated egress paths in future drive-through clinic planning.

### Qualitative comparison of Virulizer to PCR

A total of 82 participants were tested on the Virulizer platform over the two conformity studies, 79 of which were used in analysis.

### PCR *C_q_* values and quantitative results for all PCR-positive samples

The cycle of quantification (*C_q_*) values and the quantitative results for both the *ORF1a/b* and *E-gene* are reported in Table 1, below. The Virulizer RAT detected SARS-CoV-2 ranging from *C_q_* 23.89 to 30.6 for the *ORF1a/b* and *C_q_* 23.67 to 30.58 for *E-gene*. One sample was negative by Virulizer RAT and had a SARS-CoV-2 viral load for *ORF1a/b* and *E-gene* of 4.65 and 4.73 log_10_ IU mL^−1^ respectively. However, another sample was Virulizer RAT positive with a viral load of approximately one log_10_ lower (*ORF1a/b* and *E-gene* of 3.76 and 3.95 log_10_ IU mL^−1^ respectively). No positive PCR results were reported during the last conformity series.

**Table 1.** The PCR *C_q_* values for all PCR-positive detections in trials 6 and 8

Day	OFC colour	*ORF C_q_*	*ORF* log_10_ IU ml^−1^	*E-gene C_q_*	*E-gene* log_10_ IU ml^−1^	IC *C_q_*	Cobas SARS-CoV-2 result	Virulizer result	Conformity
1	Blue	26.01	5.40	25.95	5.55	33.74	Detected	Detected	True positive
1	Blue	30.60	3.76	30.58	3.95	34.10	Detected	Detected	True positive
2	Blue	26.84	5.10	27.03	5.17	33.87	Detected	Detected	True positive
2	Yellow	28.10	4.65	28.31	4.73	33.86	Detected	Not Detected	False negative
2	Blue	23.89	6.15	23.67	6.33	33.92	Detected	Detected	True positive
2	Blue	24.67	5.87	24.81	5.94	34.01	Detected	Detected	True positive
2	Blue	24.53	5.92	24.73	5.97	35.35	Detected	Detected	True positive
2	Yellow	27.70	4.80	27.87	4.88	33.99	Detected	Detected	True positive
	Min	23.89	6.15	23.67	6.33				
	Max	30.60	3.76	30.58	3.95				

*C_q_*, cycle of quantification; Dtest, daily test number; Env, envelope gene; IC, internal control; OFC, oral fluid collector; ORF1, open reading frame 1a gene; Ttest, total trial test number.

#### Qualitative comparison: blue-conversion oral fluid collectors

Of 68 participant results, a total of six (8.83%) were confirmed by SARS-CoV-2 PCR assay positive. The Virulizer platform identified all six. The resulting confusion matrix and relevant bivariate parameter values are presented in Tables2  3. When compared to the reference PCR assay, the Virulizer platform had a sensitivity of 100% (95% CI: 82.5–96.5%), specificity of 91.94% (95% CI: 82.5–96.5%) and accuracy of 92.65%. The positive predictive value (PPV) was 54.55% and negative predictive value (NPV) was 100%. A Cohen’s Kappa value was κ=0.67, consistent with a substantial agreement between Virulizer results and PCR. The Matthews Correlation Coefficient (MCC) of 0.71 indicated a moderately strong agreement between the two testing methods.

**Table 2.** Confusion matrix of SARS-CoV-2 detections by PCR and Virulizer – All OFC samples. (Data of blue-only OFC in brackets)

		PCR		
		Detected	Not Detected	Totals
Virulizer	Detected	7 (6)	5 (5)	12 (11)
	Not Detected	1 (0)	66 (57)	67 (57)
	Totals	8 (6)	71 (62)	79 (68)

**Table 3.** Calculated bivariate values of comparing PCR and Virulizer based detection of SARS-CoV-2

Parameter	All OFC	Blue OFC
Sensitivity (Recall)	87.50%	100.00%
Specificity	92.96%	91.94%
Accuracy	92.41%	92.65%
PPV (Precision)	58.33%	54.55%
NPV	98.51%	100.00%
F1 Score	70.00	70.59
Cohen’s Kappa	0.66	0.67
Interpretation	Substantial Agreement	Substantial Agreement
MCC	0.68	0.71

MCC, Matthews correlation coefficient; NPV, Negative Predictive Value; PPV, Positive Predictive Value.

#### Qualitative comparison of all indicators

The addition of the 11 participant samples whose OFC did not fully convert to blue yields a total of 79 samples, of which eight (10.13%) were confirmed SARS-CoV-2 PCR positive. The Virulizer platform identified seven matched samples. The resulting confusion matrix and relevant bivariate parameter values (Tables2  3) indicates that the Virulizer platform had a sensitivity of 87.5% (95% CI: 52.9–97.8%), a specificity of 92.96% (95% CI: 84.6–97.0%) and an accuracy of 92.41% when assessing all OFCs tested. The PPV was 58.33% and NPV was 98.51%. The Cohen’s Kappa value was κ=0.66, signifying substantial agreement between Virulizer results and SARS-CoV-2 PCR. The MCC of 0.68 was similar value to that of κ.

## Discussion

Under pandemic measures DTCs provide a suitable collection point for point-of-care test (PoCT) feasibility and conformity studies. These locations are preferable, when prior clinical assessment is not required, for safe sample collection from people isolating in the community. DTCs are thus a good means of preventing at-risk groups from exposure to potential COVID-19 positive patients presenting at walk-in collection centres located at hospitals. Use of physical distancing, PPE and specimen biocontainment by MDTC staff required entry of digital data at the point of specimen collection. Further integration of validated, technician operated, point of care test results at DTCs with laboratory information systems will improve the consistency of test results and downstream clinical or public health actions.

The CRATE study demonstrated a high sensitivity and specificity of SARS-CoV-2 detection when analysing saliva samples on the Virulizer platform, when compared with a reference molecular detection method (COBAS PCR). The NPV supports application of the Virulizer RAT performed by a trained operator as a SARS-CoV-2 screening method. In practice, the limited throughput of the current Virulizer platform argues against its application in high-traffic areas such as airports or high flow emergency response testing facilities such as large DTCs. With the end of emergency pandemic measures, there is time to reflect on what has been learned [17]. The ability of DTCs to adapt to changes in test demand while maintaining surge capacity suits them to a future public health emergency response. During MDTC operation, WA reported between 4,500 and 102 000 COVID-19 cases per week [3]. The key limiting factors to sample collection capacity during surge periods were vehicle traffic management and patient registration. Conversely, economic management became more important as testing numbers waned. The MDTC site was suitably placed due to its combination of ample vehicle queue space, proximity to a large tertiary hospital with advanced laboratory infrastructure, allowing prompt transport of samples and consumables between sites for further reflex testing. Inherent limitations of the DTC model included reduction of participant recruitment to occupants of private vehicles. Staff management needed sophisticated organisation with a staff reserve, observation of demanding workplace health and safety measures including dehydration, mask-health and extreme weather exposure in the DTC [4,18]. During MDTC operations, we encountered unexpected challenges relating to private vehicle breakdowns, frequently relating to battery issues, which led to occasional traffic disruptions and delays. To address this issue in future DTC setups, we recommend incorporating a slip-lane or overtaking capability within the tent structure and considering vehicle recovery procedures in site management protocols. Additionally, we suggest incorporating a slip-lane or overtaking capability within the tent structure. This would allow for smoother traffic flow by enabling faster-moving vehicles to bypass those requiring extended testing times or experiencing mechanical issues. Such design modifications were included when reconfiguring the MDTC set up (Fig. 4) and enhanced the efficiency of drive-through testing operations, reducing wait times and improving overall throughput. To optimize traffic flow and testing efficiency, we implemented a triage system based on vehicle occupancy. Two distinct lanes were established: a 'family lane' for vehicles with multiple occupants, and a ‘single occupancy lane’ for vehicles with only one person. This strategic division allowed for more efficient processing of vehicles based on the number of tests required per car. The family lane accommodated the longer testing times needed for multiple occupants, while the single occupancy lane maintained a faster throughput for individual testers. This triage approach significantly improved overall traffic management and reduced wait times, particularly during peak testing periods. The flexibility to adjust lane allocation based on real-time demand further enhanced the DTC’s ability to manage fluctuating testing volumes efficiently.

The main limit of the Virulizer conformity study was its small sample size, particularly the low number of positive cases, which impacted statistical robustness of our findings. The limited study size was due to a combination of declining case numbers and changes in Health Department-driven test indications during the study period. While our results showed promise, wide confidence intervals resulting from small sample size preclude definitive conclusions about the assessed test performance in larger populations.

As a point-of-care test (PoCT), the Virulizer platform had advantages over RT-PCR testing, with potential future application in regional and remote locations. Extended turnaround times attributed to centralized laboratory RT-PCR services were an obstacle to effective management of clinic patients. Outside Perth, Western Australians may have to travel more than 5 h to see a primary health care professional. These remote settings rely on PoCT, courier services, or medical transit to larger centres that can diagnose and treat patients. Rapid antigen tests were not considered suitable as a stand-alone support for medical decisions due to their lack of reliability, the vagaries of self-administration and self-reporting [9,19]. A rapid PoCT should be easy to operate and interpret without additional professional or laboratory support. Although 100% specificity is the ideal, clinicians rely on multiple data sources to reach a diagnosis, therefore the combination of the specificity (91.34%) and sensitivity (100%) of the Virulizer RAT suited it for primary health care. Positive detections were made at high and low viral loads in the set of positive samples available from the study participants. The only false-negative result during the conformity assessment series was at detectable viral load but in an OFC that failed to convert its indicator colour to blue. The blue colour conversion should thus be confirmed before continuation with the test since failure to convert to blue may indicate insufficient saliva volume and the consequent risk of false negative results. All patients were limited to a maximum of 2 min of oral fluid collection. Samples with a yellow indicator transition were recorded during processing. Further studies are needed to discern whether a set time for fluid collection is required in the day-to-day administration of these tests, or whether protocol should dictate a sample is successfully collected as soon as the OFC indicator transitions to blue regardless of time. Nevertheless, yellow indicator samples could be due to an indicator failure, or patient misreporting of acidic food or drink consumption before sample collection, which could affect both indicator conversion and analyte binding [20]. Considering the number of participants in the trial, the authors included these details in the analysis. A potential improvement to the sample treatment process would be to reduce specimen viscosity after collection, as viscosity is considered to reduce test specificity due to antigen masking [9,21].

While the Virulizer demonstrated high specificity, there were false positive results, which are uncommon in rapid antigen tests. Possible explanations include cross-reactivity with other antigens, sample contamination, non-specific binding and test timing. The tolerable false positive rate for RATs can vary with the context in which the test is used. For example, in a high prevalence setting or during a pandemic, the priority might be to quickly identify and isolate positive cases to prevent further transmission, even if that means accepting a higher false positive rate. Further investigation of potential false readings in larger studies would help optimise test performance and understand its limits in varying clinical settings. Also, while RATs miss some cases with a lower viral load, RATs are still useful for quickly identifying more infectious individuals, especially in remote and regional settings where PCR assays are not readily available, or results are delayed.

A notable limitation of the CRATE study was a falling number of participants during the conformity phase. This trend reflected national and global trends at the time of specimen collection. During conformity testing, WA recorded one third the number of positive COVID-19 cases compared to the previous month. The lengthy process required for research governance approval after research ethics approval had been granted prevented an earlier start of the trial and contributed to the reduced sample size during the conformity stage. It is also possible that pandemic fatigue impacted participant recruitment as the general public began to deprioritise SARS-CoV-2 testing [17]. The state Health Department altered testing qualification guidelines to prevent most people with positive RATs from obtaining a confirmatory PCR assay result. As at-home RAT testing increased throughout 2022, PCR testing numbers fell. The conformity series in this study was therefore limited by the ebb and flow of COVID-19 infection, including emerging variants that caused less severe disease. We acknowledge potential selection bias in our study design, particularly regarding gaining access to the Virulizer test. These plausible sources of bias were difficult to avoid during an active pandemic response and should be considered when interpreting our results. Future studies should be planned even earlier in the course of a pandemic with more systematic sampling strategies to minimize potential selection bias. This would provide a more representative assessment of rapid point of care test performance across different population subgroups and clinical scenarios.

The COVID-19 pandemic highlighted the need for versatile, rapid diagnostic tools that can simultaneously detect multiple pathogens. While the Virulizer test demonstrated promising results for SARS-CoV-2 detection, its utility in a post-pandemic world would be significantly improved by expanding its repertoire to include other respiratory viruses, particularly influenza A/B and respiratory syncytial virus (RSV). This multiplexed approach would improve test relevance and increase data collection opportunities. Beyond respiratory viruses, future iterations of similar test platforms could incorporate tests for other clinically relevant antigens or biomarkers of infections with overlapping clinical syndromes. By broadening the spectrum of detectable pathogens and biomarkers, RATs and PoCTs like the Virulizer could become useful tools in multiple healthcare settings, from primary care clinics to emergency departments.

Our proof-of-concept study demonstrates the potential for deploying novel rapid antigen testing technology, such as the Virulizer, into drive-through testing clinics during a pandemic response. In the context of our study, while the Virulizer system showed promising results, its intended use as a test-to-control tool means that its performance should be evaluated primarily in terms of its ability to rapidly identify potential cases for further confirmation and isolation, rather than as a definitive diagnostic tool for treatment decisions. Future studies with larger sample sizes across a range of viral loads will be crucial to fully understand its performance characteristics and optimal role in pandemic response strategies. This study suggests that the Virulizer system, with its simple sample collection and testing process, could be useful for point-of-care SARS-CoV-2 detection, particularly in regional and remote settings where delays from collection to actionable results can reduce the impact of clinical and public health interventions.

The drive-through clinic, coupled with rapid testing, offers a scalable and efficient approach to mass testing that could be adapted for future infectious disease outbreaks. Our experience highlights the importance of adaptive testing strategies in public health emergencies, while underscoring the challenges of conducting research during rapidly evolving pandemic conditions. In future, preparations should be made to initiate larger validation studies during the earliest stages of an epidemic. Ultimately, the lessons learned from this study will help prepare for and respond to future pandemics.

## Supplementary material

10.1099/jmm.0.001875 Uncited Table S1.

## Abbreviations

COVID-19: coronavirus infectious disease 2019
*C_q_*: cycle of quantification
CRATE: COVID Rapid Antigen Test Evaluation
DTC: drive-through testing centre
FIFO: fly-in fly-out
LIS: laboratory information system
MCC: Matthews correlation coefficient
MDTC: Murdoch drive-through testing centre
NHMRC: National Health and Medical Research Council
NPV: negative predictive value
OFC: oral fluid collector
PCR: polymerase chain reaction
(PoCT): point of care test
PPE: personal protective equipment
PPV: positive predictive value
PRS: patient registration system
RAT: rapid antigen test
SARS-CoV-2: severe acute respiratory syndrome coronavirus 2
TTIQ: testing, tracing, isolation and quarantine
UCNP: upconversion nanoparticle
WA: Western Australia
WHO: World Health Organization
