# Supplementary material for: Lessons learned: drive-through COVID-19 clinic testing during an adaptive epidemic response and a point-of-care test assessment of a computer-read rapid lateral flow immunoassay with fluorescence-based detection
Source: J Med Microbiol. 2024 Sep 2;73(9):001875. doi: 10.1099/jmm.0.001875 (PMC12455235; doi:10.1099/jmm.0.001875)
Supplement: Uncited Table S1. [file jmm-73-01875-s001.pdf]

Supplementary Table 1: Calculations used for data analysis. FN false negative, FP false positive, p probability of, p_o probability of observed agreement, p_e probability of agreement by chance, TN true negative, TP true positive.

Formulas used for data analysis	
**Parameter**	**Calculation**
Sensitivity	TP/(TP+FN)
Specificity	TN/(TN+FP)
Accuracy	(TP+TN)/(TP+TN+FP+FN)
Positive predictive value (PPV)	TP/(TP+FP)
Negative predictive value (NPV)	TN/(FN+TN)
F1 score	2*((PPV*Sensitivity)/(PPV+Sensitivity))
Cohen's kappa (κ)	$(p_o-p_e)/(1-p_e)$<br>↪ Where:<br>p_o = (TP+TN)/Total<br>p_e = pTP+pTN<br>↪ Where:<br>pTP = [(TP+FP)/TOTAL]*[(TP+FN)/TOTAL]<br>pTN = [(FN+TN)/TOTAL]*[(FP+TN)/TOTAL]
Matthews correlations coefficient (MCC)	(TP*TN-FP*FN)/[(TP+FP)(TP+FN)(TN+FP)(TN+FN)]^(1/2)

Supplementary Table 2: Interpretation of Cohen's Kappa

**κ**	**Interpretation**
0	No agreement
0.10 – 0.20	Slight agreement
0.21 – 0.40	Fair agreement
0.41 – 0.60	Moderate agreement
0.61 – 0.80	Substantial agreement
0.81 – 0.99	Near perfect agreement
1	Perfect agreement
